# Ordinance 3992/2017: challenges and advances for resource management in the Brazilian Unified Health System (SUS)

**DOI:** 10.11606/s1518-8787.2019053001052

**Published:** 2019-07-10

**Authors:** Blenda Leite Saturnino Pereira, Antonio Carlos Rosa de Oliveira, Daniel Resende Faleiros

**Affiliations:** I Conselho Nacional de Secretarias Municipais de Saúde. Brasília, DF, Brasil; II Conselho Nacional de Secretarias de Saúde. Brasília, DF, Brasil

**Keywords:** Unified Health System, economics, Financial Resources in Health, organization & administration, Healthcare Financing, Sistema Único de Saúde, economia, Recursos Financeiros em Saúde, organização & administração, Financiamento da Assistência à Saúde

## Abstract

To advance in order to overcome the challenge of enabling greater autonomy in the use of financial resources in the Unified Health System (SUS), system managers agreed that transfers from the Union to other federated entities will be carried out through a financial investment account and a costing account. Over the past few years, states and municipalities managed more than 34,000 bank accounts dedicated to the Union’s on-lendings, in which balance exceeded R$8 billion. However, from 2018, Ordinance 3,992/2017 unequivocally separated the budget flow from the financial flow, and the fund-to-fund transfers started to be carried out in only 11,190 bank accounts. Since then, managers have had financial autonomy in the management of financial resources received from the Union, if in accordance with the parameters established in their respective budget items at the end of each fiscal year.

## RESUMO

A fim de avançar na superação do desafio de viabilizar maior autonomia na utilização dos recursos financeiros no Sistema Único de Saúde, os gestores do sistema pactuaram que as transferências da União aos demais entes federados passam a ser realizadas por meio de uma conta financeira de investimento e uma conta de custeio. Ao longo dos últimos anos, estados e municípios chegaram a gerenciar mais de 34 mil contas bancárias dedicadas somente aos repasses da União, nas quais foram acumulados saldos acima de R$8 bilhões. Entretanto, a partir de 2018, a Portaria 3.992/2017 separou de forma inequívoca o fluxo orçamentário do fluxo financeiro e as transferências fundo a fundo passaram a ser realizadas em apenas 11.190 contas bancárias. Desde então, os gestores passaram a ter autonomia financeira na gestão dos recursos financeiros recebidos da União, desde que obedecidos os parâmetros estabelecidos em suas respectivas peças orçamentárias ao final de cada exercício.

## INTRODUCTION

The Unified Health System (SUS), established to guarantee health as a social right to all citizens, and a duty of the Brazilian State, has as a funder the federated entities: Union, States, Federal District and municipalities. The adequate allocation of resources is essential in the financing process, in order to provide economic and financial sustainability to all management spheres responsible for guaranteeing the right to health^1^ . Despite the implementation of public health actions and services (ASPS), the adequate and necessary financing and the necessary autonomy in the use of the financial resources received from other federated entities have not been a reality for municipal management^2^ . It is highlighted that, in Arretche’s view^3^ , autonomy is an indispensable condition for managers to exercise innovation and creativity to execute these actions.

In the past years, the values dedicated to the ASPS, transferred by the Union to the subnational entities, have registered less representation in relation to the total expenditures of the Ministry of Health. In 2014, on-lendings reached 67.0% of total expenditures of the healthcare sector, compared to 60.1% in 2017. Based on 2014, between 2015 and 2017, because of the increase of Union expenses, states and municipalities no longer received R$14.9 billion ( Table )^a^ .

**Table:** Public expenditure on public health actions and services and representativeness of the National Health Fund transfers (2014 to 2017).

Year	Federal			State			Municipal		
		
	Expenses (thousand R$)^a,c^	Sent to states and municipalities (thousand R$)^b,c^	%	Expenses (thousand R$)^a,c^	Received from the Union (thousand R$)^b,c^	%	Expenses (thousand R$)^a,c^	Received from the Union (thousand R$)^b,c^	%
2014	115,076,875	77,139,340	67.0	71,758,773	21,580,321	30.1	84,375,761	55,559,019	65.8
2015	113,409,395	74,090,892	65.3	68,620,617	20,132,504	29.3	81,862,935	53,958,390	65.9
2016	112,550,592	71,028,183	63.1	67,055,831	18,544,164	27.7	83,168,055	52,484,020	63.1
2017	118,780,816	71,349,729	60.1	70,426,625	18,742,053	26.6	84,251,486	52,607,676	62.4

Source: ^a^Sistema de Informações sobre Orçamentos Públicos em Saúde and ^b^National Health Fund. Own elaboration.

^c^ Monetary values in thousands of *Reais* updated to July 2018 according to the National Consumer Price Index of the Brazilian Institute of Geography and Statistics (R$1.25 (2014) = R$1.13 (2015) = R$1.06 (2016) = R$1.03 (2017) = R$1.00 (July 2018).

^a^ R$1.00 = USD3.8658, according to *Banco Central do Brasil* (Brazilian Central Bank) on June 11, 2019.

In 2017, the Tripartite Interagency Committee of SUS agreed that transfers of resources from the Union, starting in 2018, will be carried out through an account for the financial movement of investment resources and another account for the movement of resources destined to the costs of ASPS^4^ . The purpose of this agreement was to overcome the challenge of promoting greater autonomy to subnational entities^5^ in the use of financial resources, as well as to mitigate the effects of the system’s gross under-funding.

By 2018, funds allocated to the costing of ASPS were transferred through five financing blocks, passed through hundreds of labels. Therefore, the financial flow was linked to the budget flow, which compromised the autonomy of financial management of subnational entities. Survey conducted by the National Health Fund (FNS – *Fundo Nacional de Saúde* ) identified more than 800 different on-lendings to attend actions, programs, incentives and strategies of the Ministry of Health. This practice, known as “labeling” or “boxes”, was used to segregate resources in specific subaccounts, which made financial management difficult and induced the accumulation of balances^6^ .

To give normative support to the agreement of the SUS managers, Ordinance 3,992/2017^7^ , which alters the Consolidation Ordinance No. 6/GM/MS/2017^8^ , was published to provide for the financing and transfer of federal resources to ASPS to other federated entities, in the fund-by-fund modality, in accordance with Article 18 of Complementary Law 141/2012^9^ .

### Advances and Challenges

Ordinance 3,992/2017 modified the understanding of revoked Ordinance 204/2007^10^ on the transfer of federal resources that took place as six financing blocks. However, the changes brought about by the new rule raised doubts and misunderstandings about its operational form. For many years, the repealed standard has led managers to implement financial resources from the Union tied to the respective blocks or to specific budget linkages.

**Figure 1:**
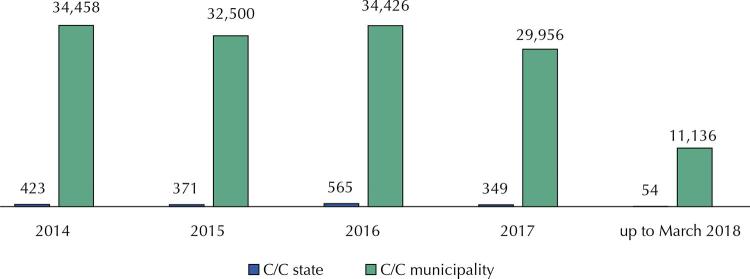
Number of checking accounts by states and municipalities that received fund-to-fund resources transfers from the Ministry of Health (2014 to March 2018).

Source: National Health Fund. Own elaboration.

The execution of these resources connected to the budget of the Union, and not to the needs of local financial flow, crystallized in the municipal management, starting to compromise the autonomy of local management. Financial execution was tied to Union programs, actions and budget plans, or even a misconception that it would be easier to manage federal resources in separate financial accounts from other transfers. Consequently, innumerable bank accounts have been opened in order to mismanagely facilitate the management of the federal funds received. Between 2014 and 2017, states and Federal District managed more than 560 accounts, and the municipalities more than 34 thousand. In 2018, after the entry into force of Ordinance 3,992/2017, there are only 54 accounts in the states and 11,136 in the municipalities: one for investment on-lending and one for costing ( Figure 1 ).

C/C: checking account

**Figure 2:**
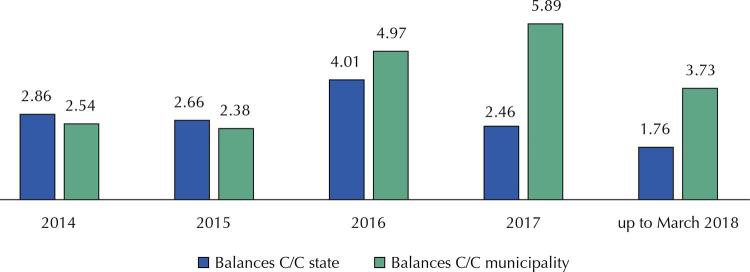
Total value of bank balances in checking accounts by states and municipalities that received fund-to-fund resources transfers from the Ministry of Health in billions of Reais (2014 to March 2018).

Source: National Health Fund. Own elaboration.

The unnecessary links induced managers and even auditors to misunderstand the analysis of tracking financial disbursements. Bank account data were used as an instrument to control budget actions, avoiding linkage to the real instruments of planning and monitoring, these ones of unequivocal accountability. More than that, many managers stopped using the received resources, to the point of annually recording billions of *Reais* accumulated in thousands of accounts. As a result, the values have accumulated in recent years, reaching, in 2017, more than R$ 8.6 billion in updated values for June 2018 ( Figure 2 ).

C/C: checking account

This diagnosis was possible due to Complementary Law 141/2012, which determines that the resources of the Union transferred to the other entities of the Federation must be moved until their final destination in specific accounts, kept in a federal official financial institution. On the other hand, the same legislation guarantees the delivery of such resources to confirmation, by the sub-national entity, the establishment and functioning of the Health Fund and the Health Council, as well as the preparation of the respective health plans. The health funds are established by laws and maintained by the direct administration of the entities, being considered budgetary units and resource managers.

In turn, Law 8,080/1990^11^ regulated the ASPS, establishing that federated entities, among other duties, prepare and periodically update the health plans and prepare the annual budget proposal, in accordance with the respective plan. It should be noted that the agreements made within the bipartite and tripartite committees reflect the municipal and regional needs within the established conditions, respecting the geographical and epidemiological specificities and the installed capacity, among others. Therefore, such agreements must be considered as part of a process that leads to the upward planning of the system, a challenge that is set to be one of the major ones for SUS^12^ consolidation.

Still as the responsibility of the federated entities, it is mentioned the elaboration of the Annual Health Schedule (PAS – *Programação Anual de Saúde* ), which dialogues directly with the Annual Budgetary Law (LOA – *Lei Orçamentária Anual* ). The PAS should contain, in a systematic way, the actions, the financial resources and other elements that contribute to achieve the objectives and the fulfillment of the plan goals, besides the annual goals for each defined action and the indicators used in the monitoring and evaluation of its execution. This whole process must be linked to the public budget, which is an instrument for planning and executing public finances. The budget as a planning instrument governs the revenue forecasting and the expenditure setting and follows strict drafting standards, becoming a legal instrument for each financial year.

The new regulation came to comply with the normative planning of the SUS regarding federal transfers without changing the calculation methodology for distribution of resources. The new rule does not create the methodology for distribution of federal resources disciplined by Article 17 Complementary Law 141/2012, it only disciplines the organization of federal transfers in two financing blocks – the Block of Costs of Health Actions and Services, and the Block of Investment of the Health Services Network – which will be transferred by the FNS, according to the existing allocation criteria.

In this context, authorized by a previous rule, the governmental expenditure of the federated entities must obey the rules imposed for its execution, that is, the requirement of resource binding is maintained. Thus, Ordinance 3,932/2017 establishes that, at the end of each fiscal year, the federal resources transferred to the subnational entities must meet the purposes defined in the Work Program of the General Union Budget, which originated the on-lendings carried out, as well as those established in the health plan and in the Annual Local Health Program.

The link between the purpose of the programs that finance the federal transfers and the application of resources by the federated entities originates in a constitutional provision (Article 167, section VI), which also prohibits “the transposition, relocation or transfer of resources of a programmed category to another or from one administrative body to another, without prior legislative authorization” (section VI). Thus, it is not possible for the Executive Power to approve the application of financial resources by another federal entity for a purpose other than that specified in the federal LOA that authorized the expense. At federal level, execution is already considered to have been effected through the transfer. Only through legislative authorization – in this case, a change of the LOA by the National Congress – it is possible to change the purpose of the expenditure foreseen in the approved capital grant for a specific budget schedule. The linking of the federal pass-through originated from a budgetary program links the purpose to this schedueling and thus corroborates the established by Complementary Law 101/2000^13^ regarding the continuity of the attachment, even in the exercise subsequent to the resource admission to the local health fund.

Failure to comply with the provisions of budgetary and financial legislation, with the consequent misuse of funds in the application of resources, implies that the manager is convicted of administrative misconduct, as provided for in Law 8,429/1992^14^ . In this case, the person responsible for the offending act shall be subjected to penalties imposed irrespective of the criminal, civil and administrative penalties provided for in the specific legislation. The public administrator is responsible for the execution of the budget according to the conduct regulated by Complementary Law 101/2000, which establishes public finance rules aimed at responsibility in fiscal management. In case of administrative misconduct, the manager will face the penalties provided for in Law 10,028/2000^15^ , which deals with crimes in public finances. Thus, at the end of each fiscal year, the financial execution should be completely adequate to the budget execution, obeying the one disciplined by Law 4,320/1964^16^ .

Ordinace 3,992/2017 unequivocally separates the budget flow from the financial flow. However, as disciplined by Law 4,320/64, at the end of each fiscal year, what is established by the budget item shall prevail. After all, the public budget is an instrument that presents to the society the intention of the manager to carry out actions throughout the year, which should be reflected in the annual management report, subsequently evaluated and approved by the local Health Board.

Therefore, it is necessary to clarify that the challenge of change, which now comes with the unification of bank current accounts, brings in its overcoming process the strengthening of planning instruments and, consequently, the management of SUS. These instruments are fundamental to the health system and essential to implement and consolidate, definitively, the tools that enable following, monitoring and evaluation actions, so commonly mentioned, but little used and appreciated over the past years. The changes arising from Ordinance No. 3,992/2017 aim to provide a better, more effective and safe application of the resources received by the Health Funds against the responsibilities of the health manager, attributed by Law 8,080/90.

## CONCLUSION

Duarte et al.^17^ report that some authors point out a possible excess of autonomy in municipal management presents itself as responsible for the fragmentation of SUS, opposing to others that identify limitations of this same autonomy when it comes to the parameters established for transfers of federal resources, mainly in the sphere of municipal management.

Ordinance 3,992/2017 did not change any linkage of financial resources destined to SUS actions and programs. The standard innovated by allowing flexibility in the financial flow of public health management, allowing the managers of the subnational spheres more autonomy in the management of financial resources received from the Union to carry out the actions agreed and programmed.

As a natural consequence of this change, the challenge of invigorating the elaboration processes, monitoring and evaluation of planning and budget instruments arises. All this process has been improving and strengthening the management and public health policy in the country. This, in turn, makes it possible for the financial resources transferred by the Union to subnational entities to be used more efficiently in the implementation of the ASPS.

It is undeniable that Ordinance 3,992/2017 contributed to overcoming the challenge of guaranteeing more autonomy to subnational entities. However, it is still far from overcoming the greater challenge: to provide the autonomy to use federal resources in order to provide means that meet the regional health needs of the population, according to local plans agreed upon and established as a guideline of Law 8,080/90.

## Footnotes

a R$1.00 = USD3.8658, according to *Banco Central do Brasil* (Brazilian Central Bank) on June 11, 2019.
